# Associations between model-predicted rivaroxaban exposure and patient characteristics and efficacy and safety outcomes in the treatment of venous thromboembolism

**DOI:** 10.1007/s11239-020-02073-z

**Published:** 2020-04-23

**Authors:** Alexander Solms, Stefan Willmann, Isabel Reinecke, Theodore E. Spiro, Gary Peters, Jeffrey I. Weitz, Wolfgang Mueck, Dirk Garmann, Stephan Schmidt, Liping Zhang, Keith A. A. Fox, Scott D. Berkowitz

**Affiliations:** 1 Clinical Pharmacometrics, Bayer AG, Berlin, Germany; 2 Clinical Pharmacometrics, Bayer AG, Wuppertal, Germany; 3 Bayer AB, Solna, Sweden; 4 Bayer U.S., LLC, Research & Development, Pharmaceuticals, 100 Bayer Boulevard, Whippany, NJ 07981 USA; 5 Janssen Research & Development, LLC, Raritan, NJ USA; 6 McMaster University and the Thrombosis & Atherosclerosis, Hamilton, ON Canada; 7 Clinical Pharmacokinetics, Bayer AG, Wuppertal, Germany; 8 Center for Pharmacometrics and Systems Pharmacology, Department of Pharmaceutics, College of Pharmacy, University of Florida, Orlando, FL USA; 9 Centre for Cardiovascular Science, The University of Edinburgh, Edinburgh, UK

**Keywords:** Drug monitoring, Exposure–response, Rivaroxaban, Venous thromboembolism

## Abstract

Anticoagulant plasma concentrations and patient characteristics might affect the benefit–risk balance of therapy. This study assessed the impact of model-predicted rivaroxaban exposure and patient characteristics on outcomes in patients receiving rivaroxaban for venous thromboembolism treatment (VTE-T) using data from the phase 3 EINSTEIN–DVT and EINSTEIN–PE studies. In the absence of measured rivaroxaban exposure, exposure estimates were predicted based on individual increases in prothrombin time (PT) and the known correlation between rivaroxaban plasma concentrations and PT dynamics. The composite efficacy outcomes evaluated were recurrent deep-vein thrombosis (DVT) and pulmonary embolism (PE) and recurrent DVT, PE and all-cause death; safety outcomes were major bleeding and the composite of major or non-major clinically relevant (NMCR) bleeding. Exposure–response relationships were evaluated using multivariate logistic and Cox regression for the twice-daily (BID) and once-daily (OD) dosing periods, respectively. Predicted rivaroxaban exposure and CrCl were significantly associated with both efficacy outcomes in the BID period. In the OD period, exposure was significantly associated with recurrent DVT and PE but not recurrent DVT, PE and all-cause death. The statistically significant exposure–efficacy relationships were shallow. Exposure–safety relationships were absent within the investigated exposure range. During both dosing periods, low baseline hemoglobin and prior bleeding were associated with the composite of major or NMCR bleeding. In conclusion, based on the underlying data and analysis, no reliable target window for exposure with improved benefit–risk could be identified within the investigated exposure range. Therefore, monitoring rivaroxaban levels is unlikely to be beneficial in VTE-T.

**Electronic supplementary material:**

The online version of this article (10.1007/s11239-020-02073-z) contains supplementary material, which is available to authorized users.

## Highlights

- Employing multivariate regression approaches, post hoc exposure–response analyses were performed using data from the EINSTEIN–DVT/EINSTEIN–PE studies to assess the impact of model-predicted rivaroxaban exposure and patient characteristics on clinical outcomes.
- Model-predicted rivaroxaban exposure–response relationships with both efficacy and safety were shallow sloped or absent within the investigated exposure range.
- Monitoring rivaroxaban levels is unlikely to be beneficial when managing venous thromboembolism treatment.

## Introduction

Rivaroxaban, an oral direct factor Xa inhibitor, is approved for several indications including venous thromboembolism (VTE) treatment (VTE-T), which includes the treatment of deep vein thrombosis (DVT) and pulmonary embolism (PE) and the prevention of recurrent DVT and PE in adults [1].

Rivaroxaban was developed to provide predictable anticoagulation with fixed-dose administration, without the need to routinely measure drug levels or perform coagulation assays for dose adjustment. This approach is supported by the high bioavailability of rivaroxaban when administered with food, and the low potential for food and drug interactions, which minimize variability in rivaroxaban exposure [2–4].

Rivaroxaban doses for VTE-T were assessed in two large multicenter phase 2 trials: ODIXa–DVT (NCT00839163) [5] and the EINSTEIN–DVT Dose-Ranging Study (NCT00395772) [6]. The EINSTEIN–DVT (NCT00440193) and EINSTEIN–PE (NCT00439777) trials [7, 8] constituted the phase 3 program that led to the approval of rivaroxaban 15 mg twice daily (BID) for the first 21 days for the initial treatment of acute DVT and PE, followed by 20 mg once daily (OD) thereafter for longer-term treatment and for the prevention of recurrent DVT and PE [1].

Advanced age and impaired renal function are associated with increased rivaroxaban exposure [1, 9], and are also independent risk factors that affect the benefits and risks (e.g. bleeding) of anticoagulant therapy. In patients receiving anticoagulants for VTE-T, factors such as previous history of VTE and concurrent cancer treatment affect the risk of VTE and/or bleeding [10–13].

Because rivaroxaban exposure varies between patients, it has been a matter of debate whether therapeutic drug monitoring (i.e., plasma-concentration-based dose adjustment) may enhance the individual benefit–risk ratio of treatment [14]. Such treatment individualization requires a robust understanding and quantification of the association between exposure and safety and efficacy. Using data from the EINSTEIN–DVT/PE studies and individually predicted rivaroxaban exposure parameters, post hoc exposure–response analyses were performed to assess the impact of rivaroxaban exposure and patient characteristics on clinical outcomes in patients receiving rivaroxaban for VTE-T. The data reported here accompany the results of a similar analysis in which the impact of rivaroxaban exposure and patient characteristics on clinical outcomes were assessed in patients receiving rivaroxaban for VTE prevention.

## Methods

### Study design

Full details of the methodology and ethical conduct of EINSTEIN–DVT/PE have been published previously [7, 8]. Briefly, 8282 patients with acute symptomatic DVT or PE, with or without DVT, were randomized to receive rivaroxaban (15 mg BID for 21 days followed by rivaroxaban 20 mg OD thereafter [mean duration of treatment 208 days]) or standard therapy for ≤ 12 months (Table 1). Two composite efficacy outcomes were evaluated in the current exposure–response analysis: recurrent DVT and fatal/non-fatal PE; and recurrent DVT, fatal/non-fatal PE and all-cause death. Major bleeding and a composite of major or non-major clinically relevant (NMCR) bleeding were evaluated as safety outcomes (Table 1). Separate analyses were performed for the BID and OD dosing periods. The BID dosing period analysis included events occurring from the first until the last day of BID dosing or until 2 days after the last BID dose (for patients continuing or not continuing into the OD period, respectively). Analysis of the OD dosing period included events occurring from the first day of OD dosing until 2 days after the last OD dose.

**Table 1:** Description of the studies and outcomes included in the exposure–response analyses

Study	EINSTEIN–DVT [7]	EINSTEIN–PE [8]
Population	Patients with acute, symptomatic DVT without symptomatic PE	Patients with acute, symptomatic PE with or without DVT
Total number of patients randomized	8282	
Pertinent exclusion criteria	Concomitant use of strong CYP3A4 inhibitors (e.g., HIV protease inhibitors or systemic ketoconazole) or strong CYP3A4 inducers like rifampicin	
Rivaroxaban dose and regimen	15 mg BID for 21 days followed by 20 mg OD for 3, 6 or 12 months at the investigator’s discretion	
Comparator dose and regimen	Standard therapy with enoxaparin 1.0 mg/kg BID and warfarin or acenocoumarol (INR 2.0–3.0)	
Maximum follow-up	12 months	
Mean treatment duration	Rivaroxaban: 208 days Standard therapy: 204 days	
Total number of patients for ER analysis	4130 (15 mg BID phase) 3953 (20 mg OD phase)	
Efficacy outcomes for ER analysis	1. Composite of recurrent DVT or fatal/non-fatal PE 2. Composite of recurrent DVT, fatal/non-fatal PE and death from any cause	
Safety outcomes for ER analysis	1. ISTH major bleeding^a^ 2. Major or NMCR^b^ bleeding	

*BID* twice daily, *CYP3A4* cytochrome P450 3A4, *DVT* deep vein thrombosis, *ER* exposure–response, *HIV* human immunodeficiency virus, *INR* international normalized ratio, *ISTH* International Society on Thrombosis and Haemostasis, *NMCR* non-major clinically relevant, *OD* once daily, *PE* pulmonary embolism

^a^ISTH major bleeding was defined as: overt bleeding associated with a decrease in hemoglobin of ≥ 2 g/dL or leading to a transfusion of ≥ 2 units of packed red blood cells or whole blood; bleeding that occurred in a critical site; or bleeding contributing to death

^b^NMCR bleeding was defined as: overt bleeding that did not meet the criteria for major bleeding but was associated with medical intervention; unscheduled contact with a physician; interruption or discontinuation of a study drug; or discomfort or impairment of activities of daily life

### Patient characteristics

Patient characteristics considered in the exposure–response evaluation (including potential risk factors for clinical outcomes) were identified a priori based on a literature review [12, 15–19] and experience in EINSTEIN–DVT/PE [7, 8]. Continuous variables, including age, were categorized to aid interpretation.

### Model-predicted rivaroxaban exposure

Because rivaroxaban plasma concentrations were not measured in the EINSTEIN studies, a previously reported integrated population pharmacokinetic (popPK) model, which was partly developed using data from the phase 2 DVT studies [5, 6, 20] was employed to predict individual rivaroxaban exposure estimates using patient characteristics known to influence rivaroxaban pharmacokinetics [21]. Trough plasma concentration (C_trough_), maximum plasma concentration (C_max_) and area under the plasma concentration–time curve from 0 to 24 h (AUC_0–24_) at steady state were predicted for each patient based on individual characteristics (age, weight, renal function measured as calculated creatinine clearance [CrCl] using the Cockcroft–Gault equation, and sex) and rivaroxaban dose. Using patient characteristics alone to predict individual rivaroxaban exposure might not adequately reflect the expected variability; therefore, a new approach, to enhance model-predicted rivaroxaban exposures based on the collateral correlation between rivaroxaban plasma concentration and measured prothrombin time, was applied as described previously [22].

Exposure–response analyses were performed for all patients who received at least one dose of rivaroxaban. For the rivaroxaban OD dosing period, relationships between exposure metrics and clinical outcomes were explored using Kaplan–Meier plots.

### Regression analyses

For the BID dosing period, exposure–response relationships were evaluated using logistic regression with application of penalized likelihood (Firth method) to avoid small-sample bias [23]. Time-to-event analysis was not expected to provide additional information in this context because the treatment duration was short (3 weeks). For the long-term OD dosing period, exposure–response relationships were analyzed using time-to-event Cox proportional regression. The analysis was conducted using R (version 3.3.0) and the logistf, survival, coxphf and pspline packages.

Relationships between rivaroxaban exposure metrics, patient characteristics and each of the efficacy and safety outcomes were quantified using the following methods. Initially, univariate regression analyses were performed using C_trough_, C_max_ or AUC_0–24_ as independent variables, assuming a linear relationship for the continuous exposure measures (logistic regression) or a linear relationship between the exposure measures and the log hazard of outcome events (Cox proportional regression). The exposure metric most strongly associated with the occurrence of an event, indicated by the lowest Akaike information criterion (AIC) value generated by the univariate analyses, was then combined with the selected patient characteristics for VTE-T as independent variables for predicting the probability of the outcomes in multivariate regression analyses (the full model). Age and CrCl were expected to influence outcomes [1], and were, therefore, forced into the models regardless of their statistical significance. This forced inclusion was done to avoid bias in the variable selection process due to confounding variables, given that a patient’s CrCl and age are also correlated with rivaroxaban exposure. Active malignancy at randomization was an additional covariate forced into the model for the efficacy and safety analyses. With selected variables forced into the model, backward elimination, based on AIC values, was performed on the other variables until no further variable was removed. All statistically non-significant variables, with the exception of the forced input variables, were removed to generate the final model. Statistical significance refers to covariates, including exposure, with a likelihood ratio test p value no greater than 0.01.

If exposure was included in the final model, hazard ratios (HRs) or odds ratios (ORs) were generated for the variables in the final models and shown in forest plots. The reference category was the most-commonly observed category for the variable, except for region, for which Western Europe was set as the reference. For exposure metrics, the median value of each dose was set as the reference to represent the typical exposure in a patient at that dose level. The final models were used to simulate the probability of efficacy or safety events versus exposure in a typical patient population (i.e., with individual patient characteristics set to reference values).

## Results

### Patient characteristics

Supplemental Table 1 shows the patient characteristics selected for evaluation. Supplemental Table 2 shows the count and proportion of patients in the EINSTEIN–DVT/PE studies with each characteristic. Among the 4130 patients included in the BID dosing period, 63% were < 65 years of age, 21% were 65–75 years of age, and 16% were > 75 years of age; 56% were male. Overall, 8% of these patients had CrCl < 50 mL/min and approximately 6% had active malignancy at randomization.

### Rivaroxaban exposure predictions and event rates

Rivaroxaban exposure data were predicted in 4130 patients who participated in the BID dosing period and in 3953 patients who participated in the OD dosing period (Supplemental Table 3). The derived, model-based exposure metrics showed moderate variability, with C_trough_ being the most variable parameter (coefficient of variation: 43.0–52.8%). The predicted exposure metrics were all highly correlated (correlation coefficient > 0.93) within a given individual. The observed efficacy and safety outcome event rates are presented in Supplemental Table 4.

### Regression analyses

Results of the final exposure–response models are summarized in Table 2.

**Table 2:** Results of the final exposure–response models

Variables	Efficacy		Safety	
	DVT/PE	DVT/PE/all-cause death	Major bleeding	Major/NMCR bleeding
BID period				
Active malignancy at randomization^a^	n.s	n.s	n.s	n.s
Age^a^	n.s	n.s	n.s	n.s
CrCl^a^	X	X	n.s	n.s
Best exposure	C_trough_	C_trough_	n.s	n.s
Other significant covariate	None	None	Baseline hemoglobin	Baseline hemoglobin, bleeding history, NSAID use
OD period				
Active malignancy at randomization^a^	n.s	X	n.s	n.s
Age^a^	n.s	n.s	n.s	n.s
CrCl^a^	n.s	X	n.s	n.s
Best exposure	C_trough_	n.s	n.s	n.s
Other significant covariate	None	None	Baseline hemoglobin, bleeding history	Baseline hemoglobin, bleeding history, clinically relevant bleeding in the BID period

*BID* twice daily, *CrCl* creatinine clearance, *C*_*trough*_ trough plasma concentration, *DVT* deep vein thrombosis, *NMCR* non-major clinically relevant, *n.s*. not significant, *NSAID* non-steroidal anti-inflammatory drug, *OD* once daily, *PE* pulmonary embolism

^a^Forced input variables

X denotes statistically significant exposure–response relationship (p ≤ 0.01)

#### Exposure–efficacy analysis

**Fig. 1:**
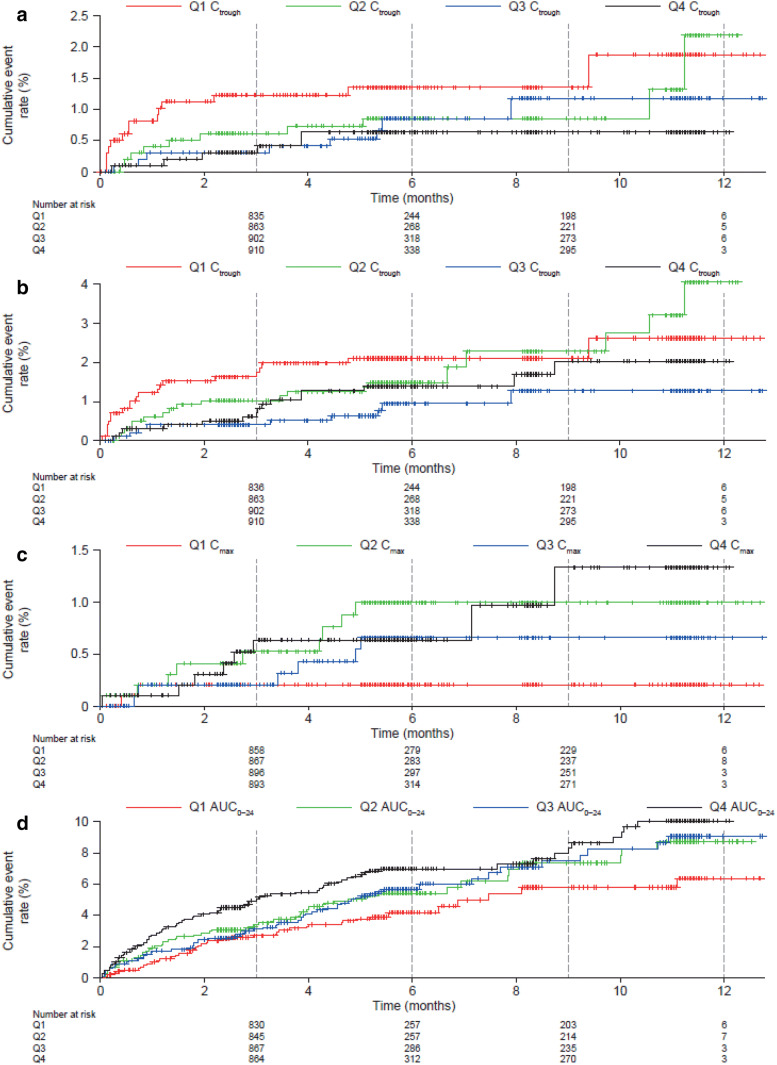
Kaplan–Meier plots of the cumulative event rate during the VTE-T OD dosing period of the composite efficacy outcomes of **a** a composite of recurrent DVT and fatal/non-fatal PE, and **b** a composite of recurrent DVT, fatal/non-fatal PE and all-cause death versus predicted rivaroxaban steady-state C_trough_; and **c** major bleeding versus predicted rivaroxaban C_max_ and **d** a composite of major or NMCR bleeding versus predicted rivaroxaban AUC_0–24_. 0 denotes the start of the OD treatment period. *AUC*_*0–24*_ area under the plasma concentration–time curve from 0 to 24 h, *C*_*max*_ maximum plasma concentration, *C*_*trough*_ trough plasma concentration, *DVT* deep vein thrombosis, *NMCR* non-major clinically relevant, *OD* once daily, *PE* pulmonary embolism, *Q *quartile, *VTE-T* venous thromboembolism treatment

In the univariate regression analysis for efficacy, C_trough_ was selected for further investigation based on AIC value (Supplemental Table 5). Cumulative event rates versus stratified C_trough_ values for the OD dosing period are shown in Kaplan–Meier plots (Fig. 1a, b). There was no apparent trend between quartiles of C_trough_ and event rates for the composite efficacy outcomes during the OD dosing period**.**

**Fig. 2:**
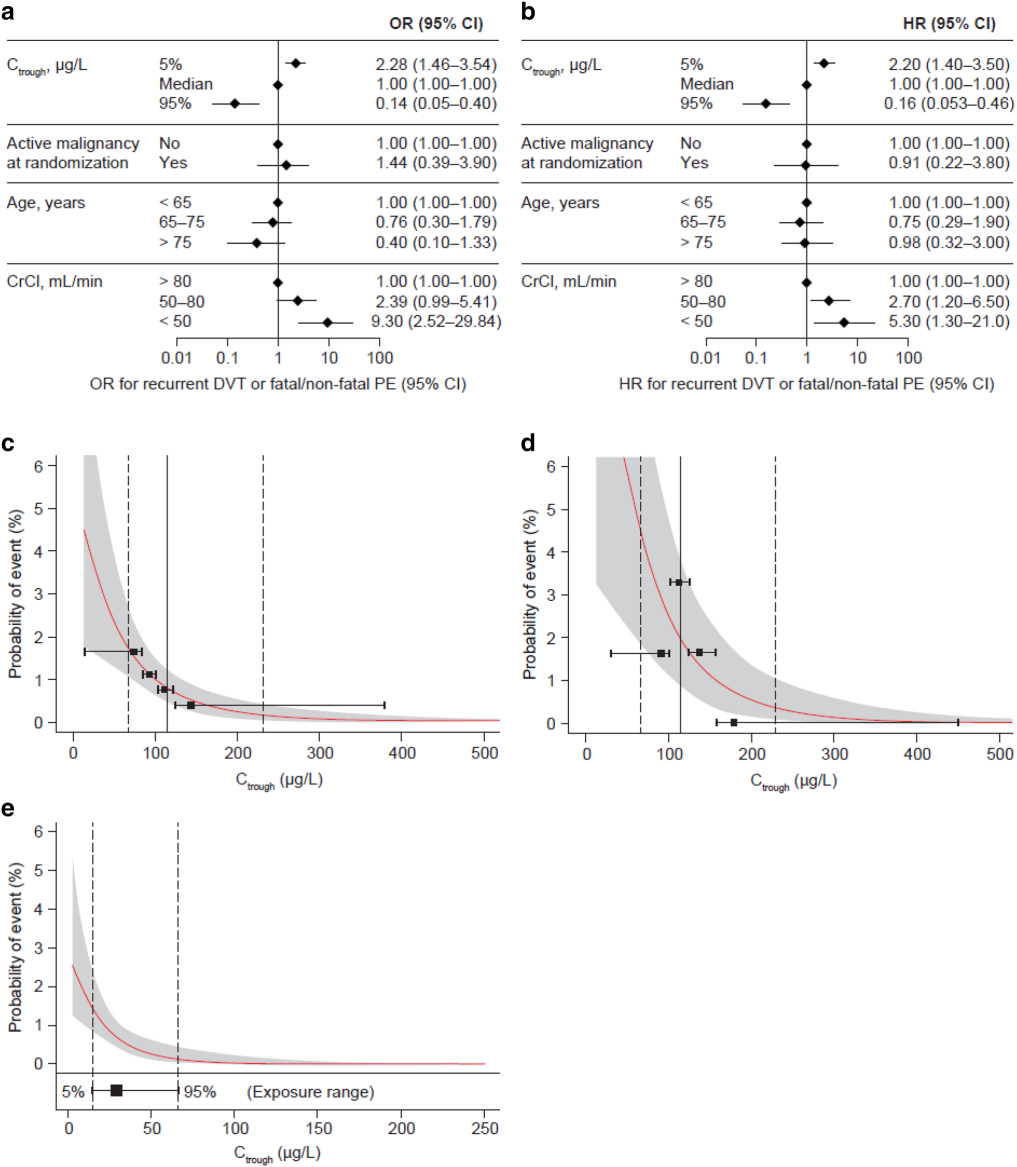
Exposure–response relationships for the composite efficacy outcome of recurrent DVT and fatal/non-fatal PE in patients receiving rivaroxaban for VTE-T.^a^**a** ORs at 21 days (BID dosing period);^a^**b** HRs at 6 months (OD dosing period);^b^**c** probability of an event at 21 days (BID dosing period) in a typical patient; and **d** probability of an event at 21 days (BID dosing period) in a typical patient with CrCl 50–80 mL/min; **e** probability of an event at 6 months (OD dosing period). Solid red lines represent predicted probability, and shaded areas represent 95% CIs. Vertical dashed lines indicate the 5th and 95th percentiles of C_trough_, and vertical solid lines indicate median C_trough_ in the study population. In **c** and **d** horizontal solid black lines represent quartiles of exposure in the reference population (no active malignancy at randomization, age < 65 years and CrCl > 80 mL/min), and black squares represent the observed fraction of events at the median of exposure within each quartile of exposure. In **e**, the horizontal solid black line represents the range from the 5th to the 95th percentile of exposure, and the black square represents the median. *BID* twice daily, *CI* confidence interval, *CrCl* creatinine clearance, *C*_*trough*_ trough plasma concentration, *DVT* deep vein thrombosis, *HR* hazard ratio, *OD* once daily, *OR* odds ratio, *PE* pulmonary embolism, *VTE-T* venous thromboembolism treatment. ^a^Among the forced variables (age, CrCl and active malignancy at randomization) in the final exposure–efficacy models, only CrCl displayed a significant association with the efficacy outcome of recurrent DVT or fatal/non-fatal PE during the BID dosing period. Results of the likelihood ratio test for the final exposure–efficacy models are shown in Supplemental Table 6. ^b^No patient characteristics, including the forced input variables of age, CrCl and active malignancy at randomization, were significantly associated with the efficacy outcome of recurrent DVT or fatal/non-fatal PE during the OD dosing period. Results of the likelihood ratio test for the final exposure–efficacy models are shown in Supplemental Table 6

The occurrence of recurrent DVT or fatal/non-fatal PE events was significantly associated with decreasing C_trough_ and CrCl during the BID dosing period (Fig. 2a) and with decreasing C_trough_ during the OD dosing period (Fig. 2b). For both the OD and BID periods, the risk of recurrent DVT or fatal/non-fatal PE decreased with increasing rivaroxaban exposure. None of the other variables investigated were significantly associated with this efficacy outcome (Supplemental Table 6).

**Fig. 3:**
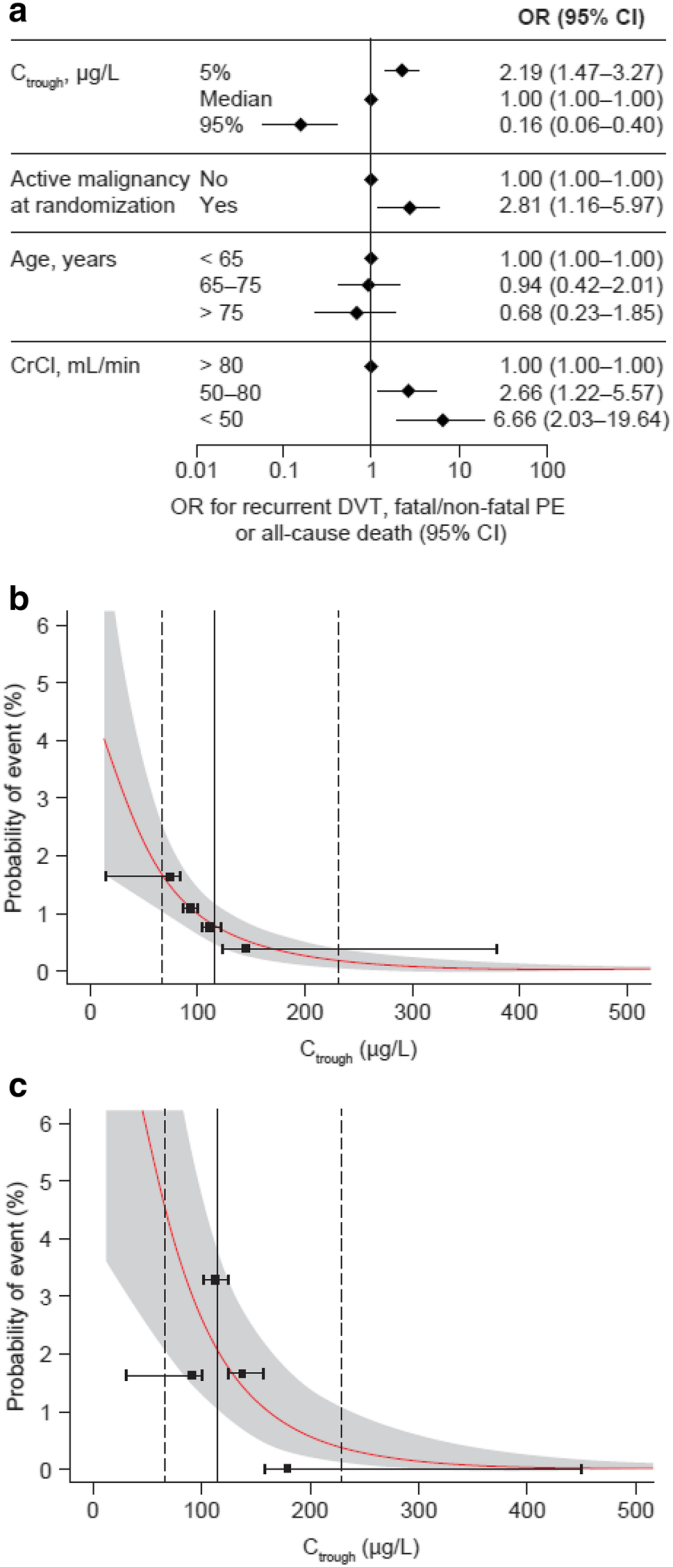
Exposure–response relationships for the composite efficacy outcome of recurrent DVT, fatal/non-fatal PE or all-cause death in patients receiving rivaroxaban for the treatment of VTE^a^ at 21 days (15 mg BID dosing period). **a** ORs at 21 days; **b** probability of an event versus C_trough_ in a typical patient; and **c** probability of an event versus C_trough_ in a typical patient with CrCl 50–80 mL/min. Solid red lines represent predicted probability, and shaded areas represent 95% CIs. Vertical dashed lines indicate the 5th and 95th percentiles of C_trough_, and vertical solid lines indicate median C_trough_ in the study population. Horizontal solid black lines represent quartiles of exposure in the reference population (no active malignancy at randomization, age < 65 years and CrCl > 80 mL/min), and black squares represent the observed fraction of events at the median of exposure within each quartile of exposure. *BID* twice daily, *CI* confidence interval, *CrCl* creatinine clearance, *C*_*trough*_ trough plasma concentration, *DVT* deep vein thrombosis, *OR* odds ratio, *PE *pulmonary embolism, *VTE* venous thromboembolism. ^a^Among the forced variables (age, CrCl and active malignancy at randomization) in the final exposure–efficacy models, only CrCl displayed a significant association with the efficacy outcome of recurrent DVT, fatal/non-fatal PE or all-cause death during the BID dosing period. Results of the likelihood ratio test for the final exposure–efficacy models are shown in Supplemental Table 6

The occurrence of recurrent DVT, fatal/non-fatal PE or all-cause death was significantly associated with decreasing C_trough_ and CrCl during the BID dosing period (Fig. 3a, Supplemental Table 6). During the OD dosing period, C_trough_ was not significantly associated with this outcome [HR 0.99 (95% confidence interval (CI) 0.97–1.00)]; however, CrCl < 50 mL/min versus > 80 mL/min [HR 4.56 (95% CI 1.74–11.95)] and active malignancy at randomization [HR 5.61 (95% CI 3.15–10.01)] were associated with a significantly increased risk of this outcome (Supplemental Table 6).

Exposure–efficacy relationships were shallow or not significant. An increase from the 5th to the 95th percentile of rivaroxaban exposure was predicted to be associated with a decrease from 1.72% (95% CI 1.08–2.71) to almost zero (95% CI 0.03–0.41) in the probability of recurrent DVT or fatal/non-fatal PE during the BID dosing period (Fig. 2c) and from 4.13% (95% CI 1.85–8.61) to < 0.5% (95% CI 0.07–1.07) in a typical patient with CrCl 50–80 mL/min (Fig. 2d). During the OD dosing period, an increase in exposure from the 5th to the 95th percentile was predicted to reduce the probability of this efficacy outcome from 1.45% (95% CI 0.87–2.43) to almost zero (95% CI 0.03–0.41) (Fig. 2e).

The probability of recurrent DVT, fatal/non-fatal PE or all-cause death was predicted to decrease from 1.62% (95% CI 1.03–2.53) to almost zero (95% CI 0.03–0.38) (Fig. 3b) and from 4.3% (95% CI 2.06–8.48) to < 0.5% (95% CI 0.09–1.09) at 21 days in a typical patient with CrCl 50–80 mL/min (Fig. 3c) as rivaroxaban exposure increased from the 5th to the 95th percentile. No significant associations between rivaroxaban exposure and this efficacy outcome were observed during the OD treatment period.

### Exposure–safety analysis

Following univariate regression analysis, C_max_ was selected for further investigation in exposure–safety analyses for the BID dosing period. For the OD period, C_max_ and AUC_0–24,_ were associated with the lowest AIC values and selected for further investigation for major bleeding and a composite of major or NMCR bleeding, respectively (Supplemental Table 5). Cumulative event rates versus stratified C_max_ and AUC_0–24_ values for the OD dosing period suggested that the cumulative event rate for major bleeding (Fig. 1c) and major or NMCR bleeding (Fig. 1d) increased as the rivaroxaban C_max_ or AUC_0–24_ value, respectively, increased.

However, in the context of a multivariate regression analysis, model-predicted exposure was not significantly associated with major bleeding or a composite of major or NMCR bleeding during either dosing period (Supplemental Table 7).

During the BID dosing period, low baseline hemoglobin (< 13 g/dL for men, < 12 g/dL for women) was significantly associated with major bleeding [OR 7.26 (95% CI 2.61–22.48)]. Low baseline hemoglobin [OR 2.61 (95% CI 1.89–3.59)], history of bleeding [OR 2.16 (95% CI 1.34–3.35)] and non-steroidal anti-inflammatory drug (NSAID) use [OR 2.24 (95% CI 1.47–3.32)] were significantly associated with major or NMCR bleeding (Supplemental Table 8).

During the OD dosing period, low baseline hemoglobin [HR 4.70 (95% CI 2.04–10.83)] and a history of bleeding [HR 4.87 (95% CI 2.02–11.75)] were significantly associated with major bleeding. Low baseline hemoglobin [HR 1.75 (95% CI 1.32–2.32)], prior clinically relevant bleeding in the BID dosing period (HR 2.65 (95% CI 1.72–4.08)] and history of bleeding [HR 2.22 (95% CI 1.53–3.23)] were significantly associated with major or NMCR bleeding (Supplemental Table 8).

## Discussion

For patients receiving rivaroxaban for VTE-T, significant but shallow exposure–efficacy relationships were observed. This finding should be interpreted with caution because the number of events was small. In this situation, characterization of the respective exposure–response relationship is relatively imprecise in cases of no or weak-to-moderate exposure–response effect size. Given that the rate of major bleeding was low and no significant exposure–response relationship was observed, there is unlikely to be a strong exposure–response relationship within the studied exposure range. These findings are consistent with an exposure–response analysis of apixaban for VTE-T, in which the number of patients with either a thromboembolic or bleeding event was small and no statistically significant relationship between exposure and clinical outcomes could be detected [24].

Taken together with the primary study efficacy and safety outcome results [7, 8], these findings support the approved, fixed-dose rivaroxaban regimen for VTE-T. They also show that patient characteristics, such as a history of bleeding, low baseline hemoglobin and NSAID use, have a substantial impact on bleeding outcomes with rivaroxaban and are important in assessing individual bleeding risk. Renal function, measured as CrCl, had a modest effect on rivaroxaban exposure in the integrated popPK model, with age and body weight having a minor influence on exposure [21]. Indeed, the European label for rivaroxaban states that a dose reduction from 20 mg OD to 15 mg OD should be considered in patients with moderate or severe renal impairment [1]. It is advised that signs or symptoms of blood loss should be promptly evaluated in patients taking concomitant aspirin, platelet aggregation inhibitors or NSAIDs [1].

Limitations of this analysis include the paucity of direct pharmacokinetic measurements and consequent use of model-predicted exposure data, which could not fully reproduce the inter-patient variability expected in a real-world patient population. The exposure–response analyses were post hoc, and the phase 3 studies included were not designed to evaluate exposure–response relationships and the impact of patient characteristics on outcomes. Finally, these analyses were based on only the approved dosing regimen for VTE-T. To draw more reliable conclusions on the utility of a therapeutic drug monitoring treatment approach, and to establish a potential treatment algorithm that is trusted to improve individual patient treatment outcome, further systematic evaluation of data and methods and correlation with clinical events in outcomes trials would be needed.

## Conclusions

In this analysis, model-predicted rivaroxaban exposure–response relationships were shallow or absent for both safety and efficacy outcomes. Based on the underlying studies, no reliable exposure target window with improved benefit–risk could be identified within the investigated exposure range and there was no evidence that the benefit–risk balance of rivaroxaban would be enhanced by implementing therapeutic drug monitoring as a routine measure [25]. These results support the approved, fixed-dose rivaroxaban regimens for VTE-T. However, as observed with other direct oral anticoagulants, evaluating patient characteristics, particularly renal function, also provides valuable information when considering treatment with rivaroxaban.

## Electronic supplementary material

Below is the link to the electronic supplementary material.

Supplementary file1 (DOCX 45 kb)
