# Supplementary material for: Associations between model-predicted rivaroxaban exposure and patient characteristics and efficacy and safety outcomes in the treatment of venous thromboembolism
Source: J Thromb Thrombolysis. 2020 Apr 23;50(1):1–11. doi: 10.1007/s11239-020-02073-z (PMC7293979; doi:10.1007/s11239-020-02073-z)
Supplement: Supplementary file 1 — Supplementary file1 (DOCX 45 kb) [file 11239_2020_2073_MOESM1_ESM.docx]

Supplemental Appendix

**Associations between model-predicted rivaroxaban exposure and patient characteristics and efficacy and safety outcomes in the treatment of venous thromboembolism**

*Journal of Thrombosis and Thrombolysis*

Alexander Solms^1^ • Stefan Willmann^2^ • Isabel Reinecke^3^ • Theodore E. Spiro^4^ • Gary Peters^5^ • Jeffrey I. Weitz^6^ • Wolfgang Mueck^7^ • Dirk Garmann^2^ • Stephan Schmidt^8^ • Liping Zhang^5^ • Keith A. A. Fox^9^ • Scott D. Berkowitz^4^

^1^Clinical Pharmacometrics, Bayer AG, Berlin, Germany

^2^Clinical Pharmacometrics, Bayer AG, Wuppertal, Germany

^3^Bayer AB, Solna, Sweden, on behalf of Bayer AG, Research & Development, Pharmaceuticals, Berlin, Germany

^4^Bayer U.S., LLC, Research & Development, Pharmaceuticals, Whippany, NJ, USA

^5^Janssen Research & Development, LLC, Raritan, NJ, USA

^6^McMaster University, and the Thrombosis & Atherosclerosis Research Institute, Hamilton, ON, Canada

^7^Clinical Pharmacokinetics, Bayer AG, Wuppertal, Germany

^8^Center for Pharmacometrics and Systems Pharmacology, Department of Pharmaceutics, College of Pharmacy, University of Florida, Orlando, FL, USA

^9^Centre for Cardiovascular Science, The University of Edinburgh, Edinburgh, UK

**Address for correspondence** Scott D. Berkowitz, MD, Bayer U.S., LLC, Clinical Development – Thrombosis, 100 Bayer Boulevard, Whippany, NJ 07981, USA

Tel.: +1 862 404 3485

E-mail: [scott.berkowitz@bayer.com](mailto:scott.berkowitz@bayer.com)

**Supplemental Table 1** Definition of patient characteristics for inclusion in the exposure–response models

| **Covariate^a^** | **Categories^b^** | **Exposure–efficacy models** | **Exposure–safety models** |
| --- | --- | --- | --- |
| Patient characteristics included in the exposure–response models regardless of significance level | | | |
| Active malignancy at randomization | Yes, no | 🗹 | 🗹 |
| Age | < 65, 65–75, > 75 years | 🗹 | 🗹 |
| Baseline renal function (CrCl) | < 50, 50–80, > 80 mL/min | 🗹 | 🗹 |
| Patient characteristics for potential inclusion in the exposure–response models | | | |
| Intended treatment duration | 3, 6, 12 months | 🗹 | 🗹 |
| Sex | Male, female | 🗹 | 🗹 |
| Body mass index | < 25, 25–< 35, ≥ 35 kg/m^2^ | 🗹 | **–** |
| Low baseline Hb  (< 13 g/dL for men and < 12 g/dL for women) | Yes, no | **–** | 🗹 |
| History of bleeding (per SMQ) | Yes, no | **–** | 🗹 |
| Baseline drug use status | | | |
| PAI or ASA use | Yes, no | **–** | 🗹 |
| NSAID use | Yes, no | **–** | 🗹 |
| Patients entering OD dosing period | | | |
| Major bleeding in 21-day BID period | Yes, no | **–** | 🗹 |
| Major or NMCR bleeding in 21-day BID period | Yes, no | **–** | 🗹 |

*ASA* acetylsalicylic acid, *BID* twice daily, *CrCl* creatinine clearance, *Hb* hemoglobin, *NMCR* non-major clinically relevant, *NSAID* non-steroidal anti‑inflammatory drug, *OD*once daily, *PAI* platelet aggregation inhibitor, *SMQ* standardized MedDRA (Medical Dictionary for Regulatory Activities) query

^a^Missing values for covariates were filled by carry forward if a previous value for a patient was unavailable or by carry backward if only a later variable was recorded. In cases where the covariate value was not recorded at any time during the study for the patient, the median value calculated from otherwise similar patients in the population dataset was used

^b^Based on current labels or clinical/statistical rationale

**Supplemental Table 2** Counts of patient characteristics for inclusion in the exposure–response models (safety and efficacy population)

| **Covariate** | **Category** | **Number of patients** | **Percent^a^** |
| --- | --- | --- | --- |
| **BID dosing period (n = 4130)** | | | |
| Age | < 65 years | 2592 | 62.8 |
|  | 65–75 years | 883 | 21.4 |
|  | > 75 years | 655 | 15.9 |
| Baseline renal function (CrCl) | > 80 mL/min | 2767 | 67.0 |
|  | 50–80 mL/min | 1034 | 25.0 |
|  | < 50 mL/min | 329 | 8.0 |
| Active malignancy at randomization | No | 3898 | 94.4 |
|  | Yes | 232 | 5.6 |
| Sex | Female | 1836 | 44.5 |
|  | Male | 2294 | 55.5 |
| Body mass index | < 25 kg/m^2^ | 1257 | 30.4 |
|  | 25–< 35 kg/m^2^ | 2447 | 59.3 |
|  | ≥ 35 kg/m^2^ | 426 | 10.3 |
| Intended treatment duration | 3 months | 332 | 8.0 |
|  | 6 months | 2457 | 59.5 |
|  | 12 months | 1341 | 32.5 |
| Low baseline Hb (< 13 g/dL for men and < 12 g/dL for women) | No | 916 | 22.2 |
|  | Yes | 3214 | 77.8 |
| History of bleeding | No | 3866 | 93.6 |
|  | Yes | 264 | 6.4 |
| PAI or ASA use | No | 3704 | 89.7 |
|  | Yes | 426 | 10.3 |
| NSAID use | No | 3749 | 90.8 |
|  | Yes | 381 | 9.2 |
| **OD dosing period (n = 3953)** | | | |
| Age | < 65 years | 2498 | 63.2 |
|  | 65–75 years | 840 | 21.3 |
|  | > 75 years | 615 | 15.6 |
| Baseline renal function (CrCl) | > 80 mL/min | 2674 | 67.6 |
|  | 50–80 mL/min | 971 | 24.6 |
|  | < 50 mL/min | 308 | 7.8 |
| Active malignancy at randomization | No | 3738 | 94.6 |
|  | Yes | 215 | 5.4 |
| Sex | Female | 1747 | 44.2 |
|  | Male | 2206 | 55.8 |
| Body mass index | < 25 kg/m^2^ | 1192 | 30.2 |
|  | 25–< 35 kg/m^2^ | 2350 | 59.5 |
|  | ≥ 35 kg/m^2^ | 411 | 10.4 |
| Intended treatment duration | 3 months | 314 | 7.9 |
|  | 6 months | 2343 | 59.3 |
|  | 12 months | 1296 | 32.8 |
| Low baseline Hb (< 13 g/dL for men and  < 12 g/dL for women) | No | 3096 | 78.3 |
|  | Yes | 857 | 21.7 |
| History of bleeding | No | 3699 | 93.6 |
|  | Yes | 254 | 6.4 |
| Major or NMCR bleeding in 21-day BID period | No | 3805 | 96.3 |
|  | Yes | 148 | 3.7 |
| Major bleeding in 21-day BID period | No | 3941 | 99.7 |
|  | Yes | 12 | 0.3 |
| PAI or ASA use | No | 3556 | 90.0 |
|  | Yes | 397 | 10.0 |
| NSAID use | No | 3590 | 90.8 |
|  | Yes | 363 | 9.2 |

*ASA* acetylsalicylic acid, *BID* twice daily, *CrCl* creatinine clearance, *Hb* hemoglobin, *NMCR* non-major clinically relevant, *NSAID* non-steroidal anti-inflammatory drug, *OD* once daily, *PAI* platelet aggregation inhibitor

^a^Percentages are subject to rounding and may not add up to 100%

**Supplemental Table 3** Rivaroxaban exposure summary (safety and efficacy population)

| **Exposure measure** | **P05** | **Median** | | **P95** | | **Mean** | | | **CV, %** |
| --- | --- | --- | --- | --- | --- | --- | --- | --- | --- |
| **BID dosing period (n = 4130)** | | | | | | | | | |
| C_trough_ (μg/L) | 66.8 | 115 | | 230 | | 127 | | | 43.0 |
| C_max_ (μg/L) | 196 | 245 | | 340 | | 253 | | | 18.9 |
| AUC_0–24_ (μg/L×h) | 3236 | 4318 | | 6686 | | 4566 | | | 25.3 |
| **OD dosing period (n = 3953)** | | | | | | | | | |
| C_trough_ (μg/L) | 14.8 | | 30.3 | | 66.8 | | 34.4 | 52.8 | |
| C_max_ (μg/L) | 183 | | 229 | | 306 | | 236 | 17.1 | |
| AUC_0–24_ (μg/L×h) | 1984 | | 2662 | | 4060 | | 2804 | 24.7 | |

*AUC_0–24_* area under the plasma concentration–time curve from 0 to 24 hours, *BID* twice daily, *C_max_* maximum plasma concentration, *C_trough_* trough plasma concentration, *CV* coefficient of variation, *OD* once daily, *P05* 5th percentile, *P95* 95th percentile

**Supplemental Table 4** Observed event rates

|  | **Patients with event/total patients (%)** | |
| --- | --- | --- |
|  | **BID dosing period** | **OD dosing period** |
| **Efficacy outcomes** | | |
| 1. Recurrent DVT or fatal/non-fatal PE | 40/4130 (0.97) | 38/3953 (0.96) |
| 1. Recurrent DVT, fatal/non-fatal PE or all-cause death | 47/4130 (1.14) | 64/3953 (1.62) |
| **Safety outcomes** | | |
| 1. Major bleeding | 16/4130 (0.39) | 25/3953 (0.63) |
| 1. Major and NMCR bleeding | 176/4130 (4.26) | 236/3953 (5.97) |

*BID* twice daily, *DVT* deep vein thrombosis, *NMCR* non-major clinically relevant, *OD* once daily, *PE* pulmonary embolism

**Supplemental Table 5** AIC values for safety and efficacy outcomes and exposure metrics resulting from univariate assessments. Selected exposure metrics (i.e., the metric with the lowest AIC value) for multivariate assessment are marked in bold

| **Outcome** | **AUC_0–24_** | **C_max_** | **C_trough_** |
| --- | --- | --- | --- |
| **BID dosing period** | | | |
| Composite outcome 1: recurrent DVT and fatal/non-fatal PE | −1.72 | −0.602 | −**8.51** |
| Composite outcome 2: recurrent DVT, fatal/non-fatal PE and all-cause death^a^ | NA | NA | NA |
| Major bleeding | 1.70 | **1.31** | 1.99 |
| Major and NMCR bleeding | −1.14 | −**3.46** | 1.38 |
| **OD dosing period** | | | |
| Composite outcome 1: recurrent DVT and fatal/non-fatal PE | 609 | 611 | **607** |
| Composite outcome 2: recurrent DVT, fatal/non-fatal PE and all-cause death^b^ | 1029 | 1,028 | **1029** |
| Major bleeding | 406 | **405** | 407 |
| Major and NMCR bleeding | **3773** | 3774 | 3774 |

*AUC_0–24_* area under the plasma concentration–time curve from 0 to 24 hours, *AIC* Akaike information criterion, *BID* twice daily, *C_max_* maximum plasma concentration, *C_trough_* trough plasma concentration, *DVT* deep vein thrombosis, *NA* not applicable, *NMCR* non-major clinically relevant, *OD* once daily, *PE* pulmonary embolism

^a^Composite outcomes 1 and 2 were tested, and only 7 additional events were included in composite outcome 2. To be consistent with composite outcome 1 and the conducted efficacy analysis in the OD period, C_trough_ was preselected as the exposure metric for multivariate assessment

^b^As expected, all three exposure metrics were highly correlated, with marginal differences. For consistency with outcome 1, C_trough_ was selected, even though C_max_ had a slightly lower AIC than C_trough_

**Supplemental Table 6** Results of the final exposure–efficacy models

|  | **OR (95% CI)** | **p value** | **p value (LRT)** |
| --- | --- | --- | --- |
| **Recurrent DVT or fatal/non-fatal PE: BID dosing period** | | | |
| C_trough_ | 0.98 (0.97–0.99) | 0.00015 | 0.00015 |
| Cancer = yes (vs. no) | 1.44 (0.39–3.90) | 0.54 | 0.54 |
| Age = 65–75 years (vs. < 65 years) | 0.76 (0.30–1.79) | 0.55 | 0.33 |
| Age > 75 years (vs. < 65 years) | 0.40 (0.10–1.33) | 0.14 |  |
| CrCl = 50–80 mL/min (vs. > 80 mL/min) | 2.39 (0.99–5.41) | 0.05 | 0.0045 |
| CrCl < 50 mL/min (vs. > 80 mL/min) | 9.30 (2.52–29.8) | 0.0015 |  |
|  | **HR (95% CI)** | **p value** | **p value (LRT)** |
| **Recurrent DVT or fatal/non-fatal PE: OD dosing period** | | | |
| C_trough_ | 0.95 (0.92–0.98) | 0.00081 | 0.00024 |
| Cancer = yes (vs. no) | 0.91 (0.22–3.82) | 0.90 | 0.90 |
| Age = 65–75 years (vs. < 65 years) | 0.75 (0.29–1.94) | 0.55 | 0.81 |
| Age > 75 years (vs. < 65 years) | 0.98 (0.32–2.99) | 0.97 |  |
| CrCl = 50–80 mL/min (vs. > 80 mL/min) | 2.74 (1.16–6.52) | 0.02 | 0.036 |
| CrCl < 50 mL/min (vs. > 80 mL/min) | 5.32 (1.33–21.37) | 0.02 |  |
|  | **OR (95% CI)** | **p value** | **p value (LRT)** |
| **Recurrent DVT, fatal/non-fatal PE or all-cause death: BID dosing period** | | | |
| C_trough_ | 0.98 (0.98–0.99) | < 0.0001 | < 0.0001 |
| Cancer = yes (vs. no) | 2.81 (1.16–5.97) | 0.02 | 0.02 |
| Age = 65–75 years (vs. age < 65 years) | 0.94 (0.42–2.01) | 0.88 | 0.75 |
| Age > 75 years (vs. age < 65 years) | 0.68 (0.23–1.85) | 0.46 |  |
| CrCl = 50–80 mL/min (vs. > 80 mL/min) | 2.66 (1.22–5.57) | 0.014 | 0.0042 |
| CrCl < 50 mL/min (vs. > 80 mL/min) | 6.66 (2.03–19.64) | 0.0025 |  |
|  | **HR (95% CI)** | **p value** | **p value (LRT)** |
| **Recurrent DVT, fatal/non-fatal PE or all-cause death: OD dosing period** | | | |
| C_trough_ | 0.99 (0.97–1.00) | 0.10 | 0.08^a^ |
| Cancer = yes (vs. no) | 5.61 (3.15–10.01) | 0 | 0 |
| Age = 65–75 years (vs. < 65 years) | 0.58 (0.28–1.18) | 0.13 | 0.30 |
| Age > 75 years (vs. < 65 years) | 0.76 (0.35–1.66) | 0.50 |  |
| CrCl = 50–80 mL/min (vs. > 80 mL/min) | 2.82 (1.48–5.38) | 0.0017 | 0.0026 |
| CrCl < 50 mL/min (vs. > 80 mL/min) | 4.56 (1.74–11.95) | 0.0021 |  |

*BID* twice daily, *CI* confidence interval, *CrCl* creatinine clearance, *C_trough_* trough plasma concentration, *DVT* deep vein thrombosis, *HR* hazard ratio, *LRT* likelihood ratio test, *OD* once daily, *OR* odds ratio, *PE* pulmonary embolism

^a^C_trough_ was included in the final model for consistency with data from the BID dosing period

**Supplemental Table 7** Results of the full exposure–safety models: outcomes for which rivaroxaban exposure was excluded from the final model

|  | **Coefficient log(OR)** | **LLCI (20.5%)** | **ULCI (97.5%)** | **p value** |
| --- | --- | --- | --- | --- |
| **Major bleeding: BID dosing period** | | | | |
| Exposure | | | | |
| C_max_ | 0.00452061 | –0.0092302 | 0.01284657 | 0.45693988 |
| Forced into ER | | | | |
| Active malignancy at randomization = yes | 0.91848682 | –0.4935267 | 2.08874379 | 0.18296011 |
| Age = 65–75 years | 0.13899386 | –1.1044265 | 1.29857535 | 0.81885807 |
| Age > 75 years | –1.1986945 | –3.5858556 | 0.60414218 | 0.20393333 |
| CrCl = 50–80 mL/min | 0.18940026 | –1.0945491 | 1.41484931 | 0.7645521 |
| CrCl < 50 mL/min | –0.1115877 | –2.6802004 | 1.86075739 | 0.91921956 |
| Potential covariates | | | | |
| Intended treatment duration = 6 months | –0.467058 | –1.8311333 | 0.632645 | 0.42509262 |
| Intended treatment duration = 12 months | –0.1359068 | –2.3599117 | 1.30445669 | 0.87384517 |
| Sex = male | 1.04727616 | 0.01260097 | 2.26150811 | 0.04716702 |
| Low baseline Hb (< 13 g/dL for men and < 12 g/dL for women) = yes | 1.74179776 | 0.70277932 | 2.88610895 | 0.00099766 |
| History of bleeding = yes | 0.85825805 | –0.5167802 | 1.96313093 | 0.19778786 |
| PAI or ASA use = yes | 0.29292352 | –1.3591062 | 1.53089698 | 0.6879514 |
| NSAID use = yes | 0.69827753 | –0.6641073 | 1.7874834 | 0.28217244 |

|  | **HR  estimate** | **LLCI  (2.5%)** | **ULCI (97.5%)** | **p value** |
| --- | --- | --- | --- | --- |
| **Major bleeding: OD dosing period** | | | | |
| Exposure | | | | |
| C_max_ | 1.00315 | 0.99112 | 1.01533 | 0.60937 |
| Forced into ER | | | | |
| Active malignancy at randomization = yes | 1.59323 | 0.51173 | 4.96037 | 0.42152 |
| Age = 65–75 years | 2.38176 | 0.81619 | 6.9503 | 0.11223 |
| Age > 75 years | 2.91772 | 0.7824 | 10.8807 | 0.11081 |
| CrCl > 80 mL/min | 1 | NA | NA | NA |
| CrCl = 50–80 mL/min | 0.85724 | 0.29626 | 2.48044 | 0.77629 |
| CrCl < 50 mL/min | 0.4005 | 0.06357 | 2.52315 | 0.32985 |
| Potential covariates | | | | |
| Sex = female | 0.71719 | 0.30459 | 1.6887 | 0.44678 |
| Intended treatment duration = 3 months | 1.17492 | 0.14714 | 9.38167 | 0.87913 |
| Intended treatment duration = 12 months | 0.93436 | 0.37595 | 2.32218 | 0.8838 |
| Low baseline Hb (< 13 g/dL for men and < 12 g/dL for women) = yes | 4.44386 | 1.89466 | 10.42294 | 0.00061 |
| NSAID use = yes | 1.70353 | 0.56291 | 5.1554 | 0.34575 |
| PAI or ASA use = yes | 0.83995 | 0.2444 | 2.88673 | 0.78185 |
| Major or NMCR bleeding in  21-day BID period = yes | 1.52527 | 0.3368 | 6.90746 | 0.58382 |
| Major bleeding in 21-day BID period = yes | 5.0677 | 0.43127 | 59.54809 | 0.19672 |
| History of bleeding = yes | 4.79767 | 1.96414 | 11.71895 | 0.00058 |

|  | **Coefficient log(OR)** | **LLCI  (2.5%)** | **ULCI (97.5%)** | **p value** |
| --- | --- | --- | --- | --- |
| **Major and NMCR bleeding: BID dosing period** | | | | |
| Exposure | | | | |
| C_max_ | 0.00138873 | –0.0027302 | 0.00508334 | 0.49366837 |
| Forced into ER | | | | |
| Active malignancy at randomization = yes | 0.47296041 | –0.0699817 | 0.96769192 | 0.08567911 |
| Age = 65–75 years | 0.00366018 | –0.4267642 | 0.42058199 | 0.98647126 |
| Age > 75 years | –0.2572959 | –0.8104414 | 0.28158227 | 0.35213737 |
| CrCl = 50–80 mL/min | 0.24094716 | –0.1886633 | 0.66389604 | 0.26966949 |
| CrCl < 50 mL/min | 0.19481552 | –0.515155 | 0.87624903 | 0.58433399 |
| Potential covariates | | | | |
| Intended treatment duration = 6 months | –0.2219926 | –0.5881542 | 0.12884623 | 0.21796628 |
| Intended treatment duration = 12 months | 0.33175175 | –0.187607 | 0.80473959 | 0.20214254 |
| Sex = male | 0.35642554 | 0.04303412 | 0.67268675 | 0.02578128 |
| Low baseline Hb (< 13 g/dL for men and < 12 g/dL for women) = yes | 0.8733798 | 0.54825651 | 1.19480505 | 2.30E­–07 |
| History of bleeding = yes | 0.74393661 | 0.26454275 | 1.18451073 | 0.003031 |
| PAI or ASA use = yes | 0.55227481 | 0.11742993 | 0.96021386 | 0.01369411 |
| NSAID use = yes | 0.77289853 | 0.35280422 | 1.16597343 | 0.0004761 |

|  | **HR  estimate** | **LLCI  (2.5%)** | **ULCI (97.5%)** | **p value** |
| --- | --- | --- | --- | --- |
| **Major and NMCR bleeding: OD dosing period** | | | | |
| Exposure | | | | |
| AUC_0–24_ | 1.00023 | 1.00003 | 1.00044 | 0.0248 |
| Forced into ER | | | | |
| Active malignancy at randomization = yes | 1.12585 | 0.67541 | 1.87669 | 0.64934 |
| Age = 65–75 years | 1.08933 | 0.76208 | 1.55712 | 0.63878 |
| Age > 75 years | 1.35983 | 0.85898 | 2.1527 | 0.18972 |
| CrCl = 50–80 mL/min | 0.83405 | 0.57314 | 1.21374 | 0.34314 |
| CrCl < 50 mL/min | 0.72067 | 0.39477 | 1.31561 | 0.2861 |
| Potential covariates | | | | |
| Sex = female | 0.95305 | 0.73148 | 1.24174 | 0.72169 |
| Intended treatment duration = 3 months | 0.84383 | 0.40814 | 1.74463 | 0.64681 |
| Intended treatment duration = 12 months | 1.09395 | 0.81756 | 1.46378 | 0.54564 |
| Low baseline Hb (< 13 g/dL for men and < 12 g/dL for women) = yes | 1.73107 | 1.29865 | 2.30748 | 0.00018 |
| NSAID use = yes | 1.46462 | 1.00191 | 2.14104 | 0.04886 |
| PAI or ASA use = yes | 1.28649 | 0.88936 | 1.86094 | 0.18107 |
| Major or NMCR bleeding in  21-day BID period = yes | 2.43476 | 1.54541 | 3.83592 | 0.00012 |
| Major bleeding in 21-day BID period = yes | 1.0309 | 0.23956 | 4.43623 | 0.9674 |
| History of bleeding = yes | 2.14119 | 1.47165 | 3.11534 | 7.00E–05 |

*ASA* acetylsalicylic acid, *AUC_0–24_* area under the plasma concentration–time curve from 0 to 24 hours, *BID* twice daily, *C_max_* maximum plasma concentration, *CrCl* creatinine clearance, *ER* exposure–response, *Hb* hemoglobin, *HR* hazard ratio, *LLCI* lower limit of confidence interval, *NA* not applicable, *NMCR* non-major clinically relevant, *OD* once daily, *OR* odds ratio, *NSAID* non-steroidal anti-inflammatory drug, *PAI* platelet aggregation inhibitor, *ULCI* upper limit of confidence interval

**Supplemental Table 8** Results of the final exposure–safety models

|  | **OR (95% CI)** | **p value** | **p value (LRT)** |
| --- | --- | --- | --- |
| **Major bleeding: BID dosing period** | | | |
| Active malignancy at randomization = yes (vs. no) | 2.14 (0.53–6.78) | 0.26 | 0.26 |
| Age = 65–75 years (vs. < 65 years) | 1.16 (0.33–3.66) | 0.80 | 0.32 |
| Age > 75 years (vs. < 65 years) | 0.31 (0.03–1.86) | 0.21 |  |
| CrCl = 50–80 mL/min (vs. > 80 mL/min) | 1.44 (0.41–4.61) | 0.56 | 0.83 |
| CrCl < 50 mL/min (vs. > 80 mL/min) | 1.45 (0.13–8.22) | 0.72 |  |
| Low baseline Hb (< 13 g/dL for men and < 12 g/dL for women) = yes (vs. no) | 7.26 (2.61–22.48) | 0.00016 | 0.00016 |
|  | **HR (95% CI)** | **p value** | **p value (LRT)** |
| **Major bleeding: OD dosing period** | | | |
| Active malignancy at randomization = yes (vs. no) | 1.71 (0.57–5.19) | 0.34 | 0.37 |
| Age = 65–75 years (vs. < 65 years) | 2.43 (0.88–6.77) | 0.09 | 0.15 |
| Age > 75 years (vs. < 65 years) | 3.00 (0.87–10.38) | 0.08 |  |
| CrCl = 50–80 mL/min (vs. > 80 mL/min) | 0.88 (0.32–2.42) | 0.81 | 0.60 |
| CrCl < 50 mL/min (vs. > 80 mL/min) | 0.44 (0.08–2.44) | 0.35 |  |
| Low baseline Hb (< 13 g/dL for men and < 12 g/dL for women) = yes (vs. no) | 4.70 (2.04–10.83) | 0.00027 | 0.00027 |
| History of bleeding = yes (vs. no) | 4.87 (2.02–11.75) | 0.00042 | 0.0021 |
|  | **OR (95% CI)** | **p value** | **p value (LRT)** |
| **Major and NMCR bleeding: BID dosing period** | | | |
| Active malignancy at randomization = yes (vs. no) | 1.46 (0.85–2.39) | 0.17 | 0.17 |
| Age = 65–75 years (vs. < 65 years) | 1.04 (0.68–1.56) | 0.87 | 0.74 |
| Age > 75 years (vs. < 65 years) | 0.85 (0.49–1.43) | 0.54 |  |
| CrCl = 50–80 mL/min (vs. > 80 mL/min) | 1.41 (0.93–2.11) | 0.10 | 0.22 |
| CrCl < 50 mL/min (vs. > 80 mL/min) | 1.50 (0.79–2.77) | 0.21 |  |
| Low baseline Hb (< 13 g/dL for men and < 12 g/dL for women) = yes (vs. no) | 2.61 (1.89–3.59) | < 0.00001 | < 0.00001 |
| History of bleeding = yes (vs. no) | 2.16 (1.34–3.35) | 0.0021 | 0.0021 |
| NSAID use = yes (vs. no) | 2.24 (1.47–3.32) | 0.00027 | 0.00027 |
|  | **HR (95% CI)** | **p value** | **p value (LRT)** |
| **Major and NMCR bleeding: OD dosing period** | | | |
| Active malignancy at randomization = yes (vs. no) | 1.08 (0.65–1.80) | 0.75 | 0.76 |
| Age = 65–75 years (vs. < 65 years) | 1.22 (0.86–1.73) | 0.27 | 0.16 |
| Age > 75 years (vs. < 65 years) | 1.55 (0.99–2.42) | 0.05 |  |
| CrCl = 50–80 mL/min (vs. > 80 mL/min) | 0.91 (0.64–1.31) | 0.63 | 0.89 |
| CrCl <50 mL/min (vs. > 80 mL/min) | 0.92 (0.53–1.59) | 0.76 |  |
| Low baseline Hb (< 13 g/dL for men and < 12 g/dL for women) = yes (vs. no) | 1.75 (1.32–2.32) | 0.00012 | 0.00019 |
| History of bleeding = yes (vs. no) | 2.22 (1.53–3.23) | 0.00002 | 0.00012 |
| Major or NMCR bleeding in 21-day BID period = yes (vs. no) | 2.65 (1.72–4.08) | < 0.0001 | < 0.0001 |

*BID* twice daily, *CI* confidence interval, *CrCl* creatinine clearance, *C_trough_* trough plasma concentration, *Hb* hemoglobin, *HR* hazard ratio, *LRT* likelihood ratio test, *NMCR* non-major clinically relevant, *NSAID* non-steroidal anti-inflammatory drug, *OD* once daily, *OR* odds ratio
